# Supplementary material for: Exercise-induced histone lactylation in monocyte-derived macrophages restores cardiac immune homeostasis and function in sepsis-induced cardiomyopathy
Source: Nat Commun. 2025 Dec 15;17:756. doi: 10.1038/s41467-025-67443-8 (PMC12819526; doi:10.1038/s41467-025-67443-8)
Supplement: Supplementary file 1 — Supplementary Information [file 41467_2025_67443_MOESM1_ESM.pdf]

Supplementary Information

Exercise-Induced Histone Lactylation in Monocyte-Derived Macrophages Restores Cardiac Immune Homeostasis and Function in Sepsis-Induced Cardiomyopathy

Shuo Sun[1,6,7#], Chaojie Lai[1#], Chengchen Huang[1], Xinrong Ren[2], Tingyu Zhang[1], Juan Zou[3], Yiping Tong[2], Qingyan Zhou[1], Jiangting Lu[1], Zhida Shen[1], Wentao Chen[1], Ruilin Wang[1], Nikola Rabrenovic[1], Xingwu Wang[3], Boxuan Ma[1*], Junbin Qian[4,5*], Guosheng Fu[1*], Min Shang[1,2*]

1. Zhejiang Key Laboratory of Cardiovascular Intervention and Precision Medicine, Engineering Research Center for Cardiovascular Innovative Devices of Zhejiang Province, Department of Cardiology, Sir Run Run Shaw Hospital, School of Medicine, Zhejiang University, Hangzhou, 310016, China; **2.** School of Basic Medical Sciences and Forensic Medicine, Hangzhou Medical College, Hangzhou, Zhejiang, China; **3.** Laboratory of Cell fate and Metabolic regulation, Shenzhen campus of Sun Yat-sen University, Shenzhen, 518107, China; **4.** Zhejiang Key Laboratory of Precision Diagnosis and Therapy for Major Gynecological Diseases, Women's Hospital, Zhejiang University School of Medicine, Hangzhou, 310058, China; **5.** Zhejiang Provincial Clinical Research Center for Child Health, Hangzhou, 310058, China; **6.** Present address: Jining Medical University, Jining, 272067, China; **7.** Present address: School of Life Sciences, Jining Medical University, Rizhao, 276826, China

Contents:

Figure S1-14

Table S1-3

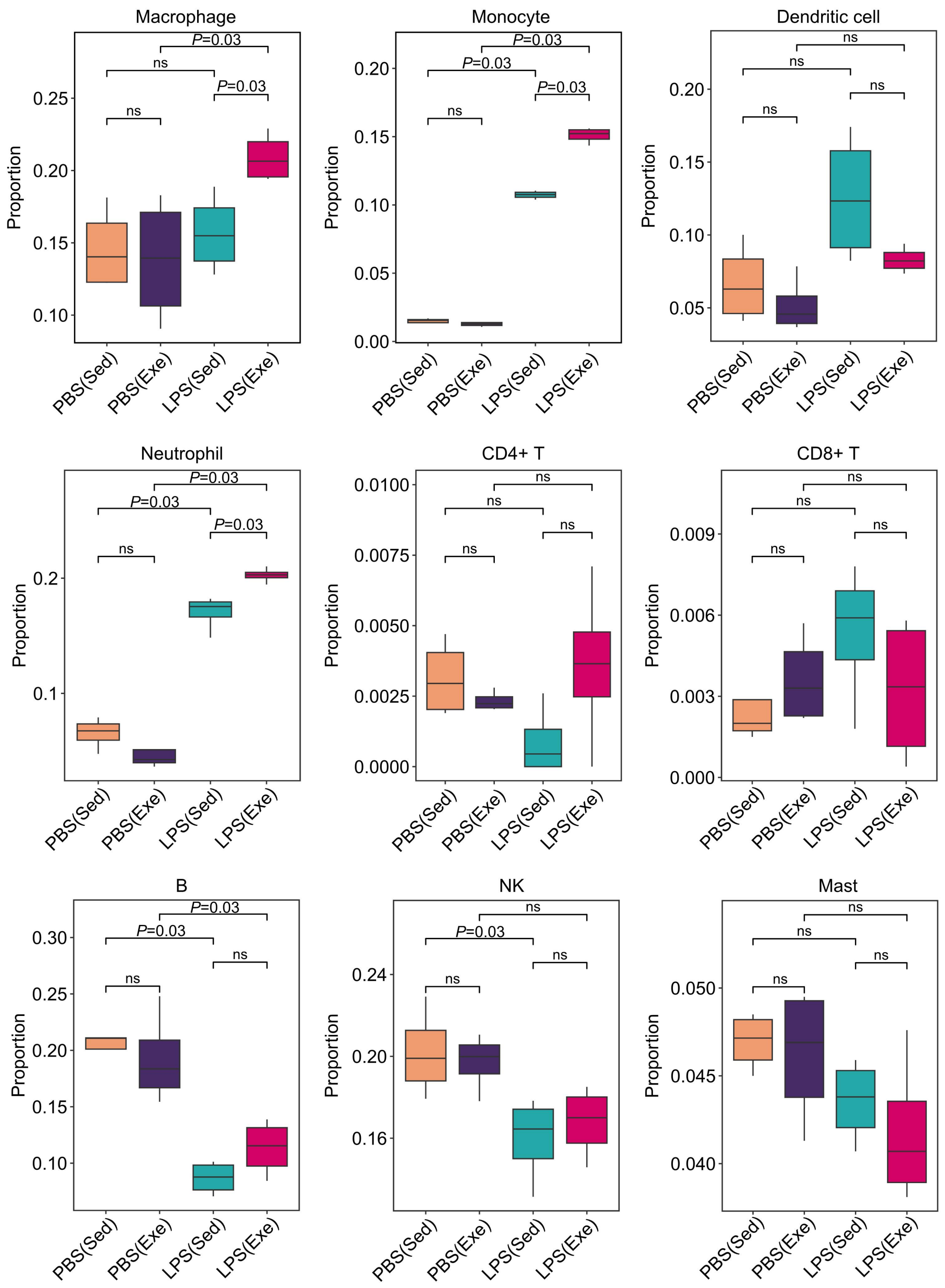

Figure S1. Box plot of immune cell proportions in different groups of mice based on bulk RNA-seq immune infiltration analysis. Number of samples included in the analysis: PBS (Sed), n=4; PBS (Exe), n=4; LPS (Sed), n=4; LPS (Exe), n=4. Data are represented as mean ± SEM. The lower whisker, lower hinge, box center, upper hinge, and upper whisker represent the minimum, lower quartile, median, upper quartile. Unpaired Mann-Whitney test was used to determine the statistical significance. ns, not significant. Source data is provided in the Source Data file.

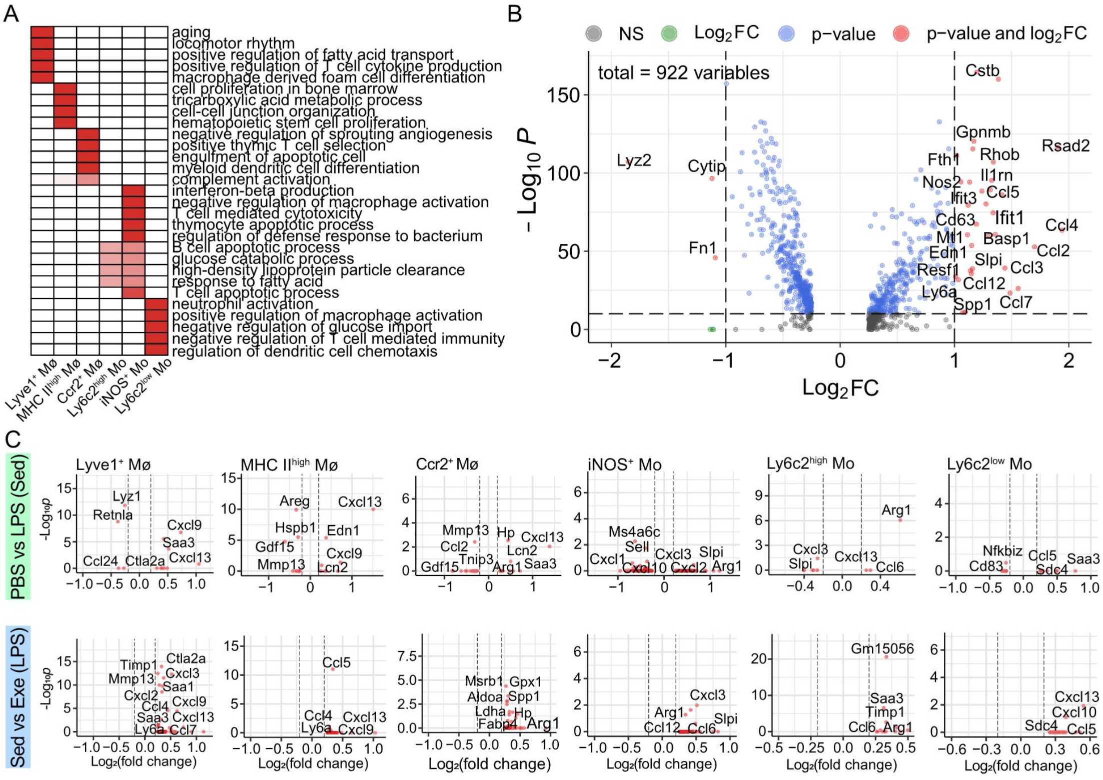

Figure S2. Gene expression characteristics of each monocyte-macrophage subsets.

A, Unique GO enrichment terms for highly expressed genes in each monocyte-macrophage subset compared to other subsets.

B, Volcano plot illustrating DEGs between the iNOS^{+} Mo and the Ly6c2^{high} Mo.

C, Volcano plot showing DEGs among monocyte-macrophage subsets between PBS (Sed) and LPS (Sed), as well as LPS (Sed) and LPS (Exe).

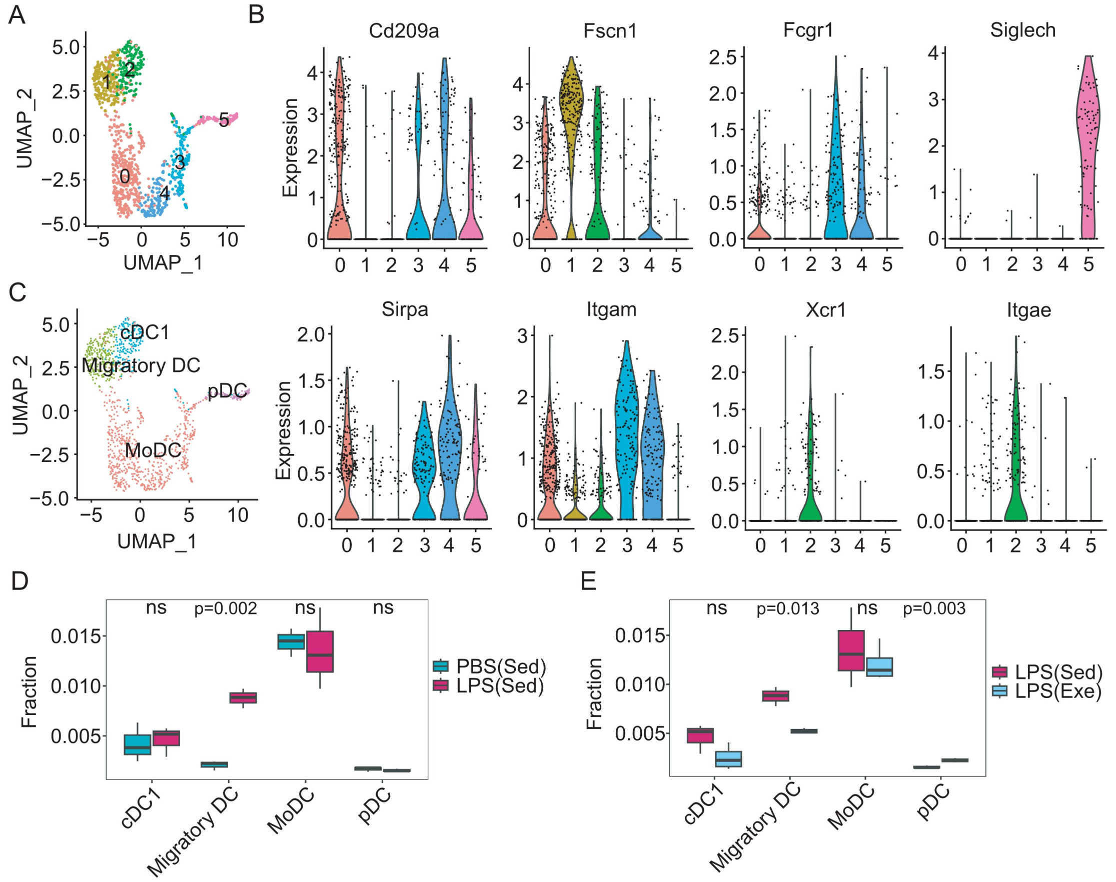

Figure S3. Identification and fraction statistics of DC subsets.

A, UMAP plot of 1,221 DCs allocated into six clusters.

B, Violin plot displaying signature gene expression for each cluster.

C, UMAP plot of four annotated DC subsets.

D-**E**, Box plot showing fraction differences of DC subsets between PBS (Sed) and LPS (Sed) (**D**) and LPS (Sed) and LPS (Exe) (**E**). Number of samples included in the analysis: PBS (Sed), n=3; LPS (Sed), n=3; LPS (Exe), n=4. Data are represented as mean ± SEM. The lower whisker, lower hinge, box center, upper hinge, and upper whisker represent the minimum, lower quartile, median, upper quartile. Unpaired two-tailed t-test was used to determine the statistical significance. ns, not significant. Source data for **D** and **E** are provided in the Source Data file.

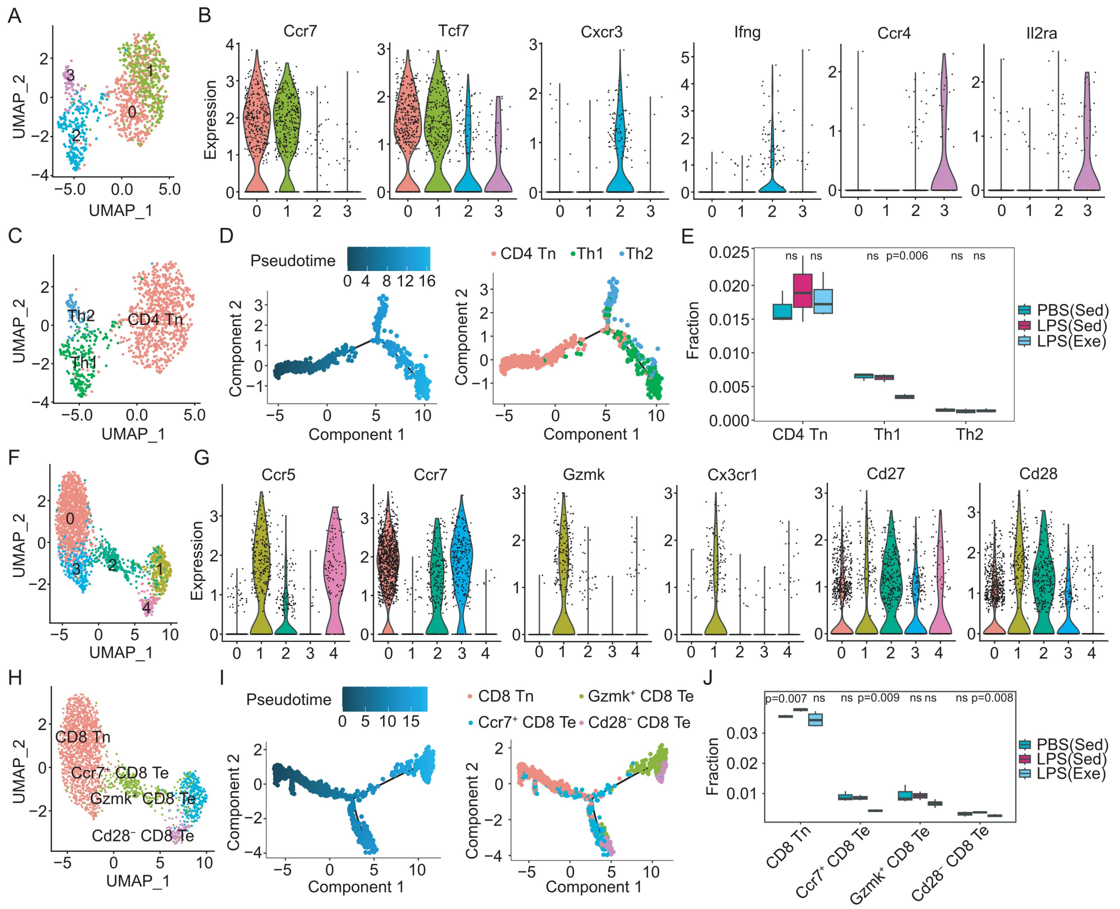

Figure S4. Identification and fraction statistics of T cell subsets.

A, UMAP plot of 1,049 CD4$^+$ T cells distributed into four clusters.

B, Violin plot of signature gene expression for each CD4$^+$ T cell cluster.

C, UMAP plot of three annotated CD4$^+$ T cell subsets.

D, Focused Monocle trajectory analysis including CD4 Tn, Th1 and Th2 cells.

E, Box plot of the fraction differences of CD4$^+$ T cell subsets across different groups.

F, UMAP plot of 2,098 CD8+ T cells distributed into five clusters.

G, Violin plot of signature gene expression for each CD8$^+$ T cell cluster.

H, UMAP plot of four annotated CD8$^+$ T cell subsets.

I, Focused Monocle trajectory analysis including CD8 Tn, Ccr7$^+$ CD8 Te, Gzmk$^+$ CD8 Te and Cd28$^-$ CD8 Te.

J, Box plot of fraction differences of CD8$^+$ T cell subsets across different groups.

Number of samples included in the analysis: PBS (Sed), n=3; LPS (Sed), n=3; LPS (Exe), n=4. Data are represented as mean ± SEM. Regarding the box polts of **E** and **J**, The lower whisker, lower hinge, box center, upper hinge, and upper whisker represent the minimum, lower quartile, median, upper quartile. Unpaired two-tailed t-test was used to determine the statistical significance. ns, not significant. Source data for **E** and **J** are provided in the Source Data file.

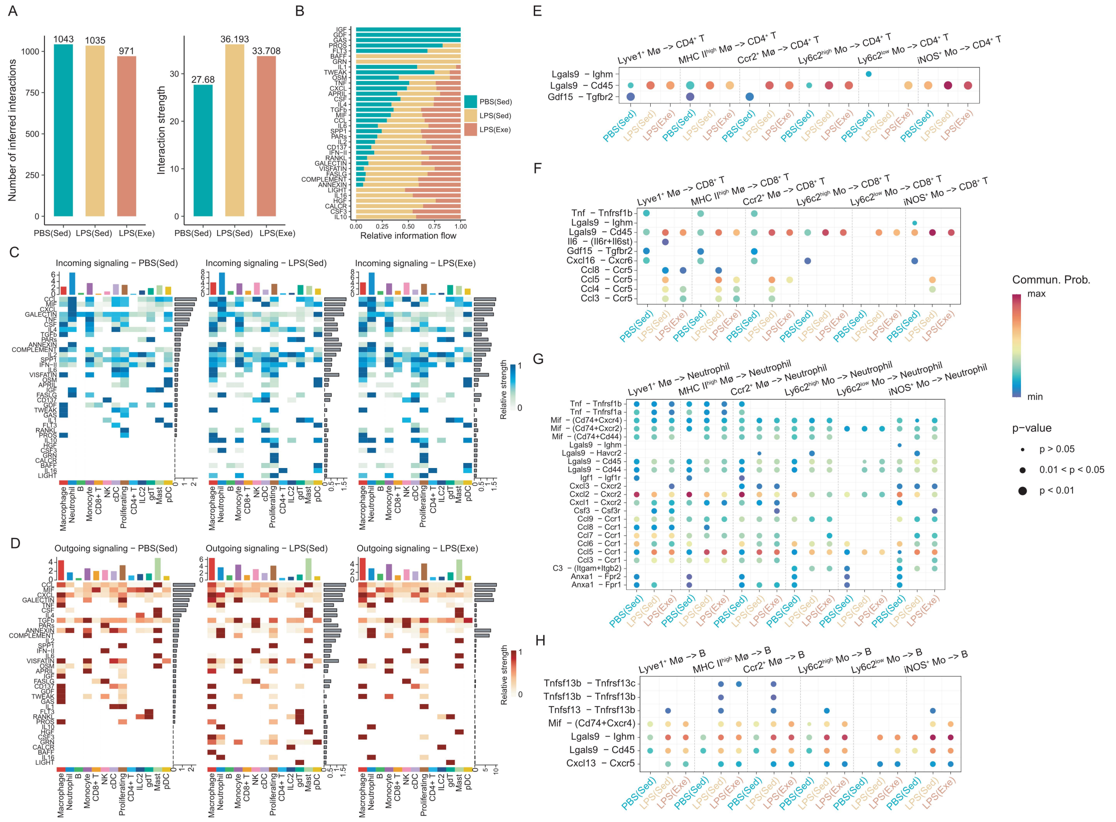

Figure S5. Global analysis of cell-to-cell communications in PBS (Sed), LPS (Sed), and LPS (Exe) groups.

A, Bar graphs showing the numbers (left) and strengths (right) of interactions in each group.

B, Stacked bar chart illustrating inferred differences in overall information flow among the three groups, with different colored signaling pathways representing enrichment in different groups.

C-D, Heatmap of incoming (**C**) and outgoing (**D**) communication probabilities for different cell subsets in each group.

E-H, Comparison of significant ligand-receptor pairs among PBS (Sed), LPS (Sed), and LPS (Exe) groups, contributing to signaling from each monocyte and macrophage subpopulation to $CD4^{+}$ T cells (**E**), $CD8^{+}$ T cells (**F**), Neutrophils (**G**) and B cells (**H**). Dot color reflects communication probabilities and dot size represents computed P-values. Empty spaces indicate zero communication probability. P-values are obtained from one-sided permutation test.

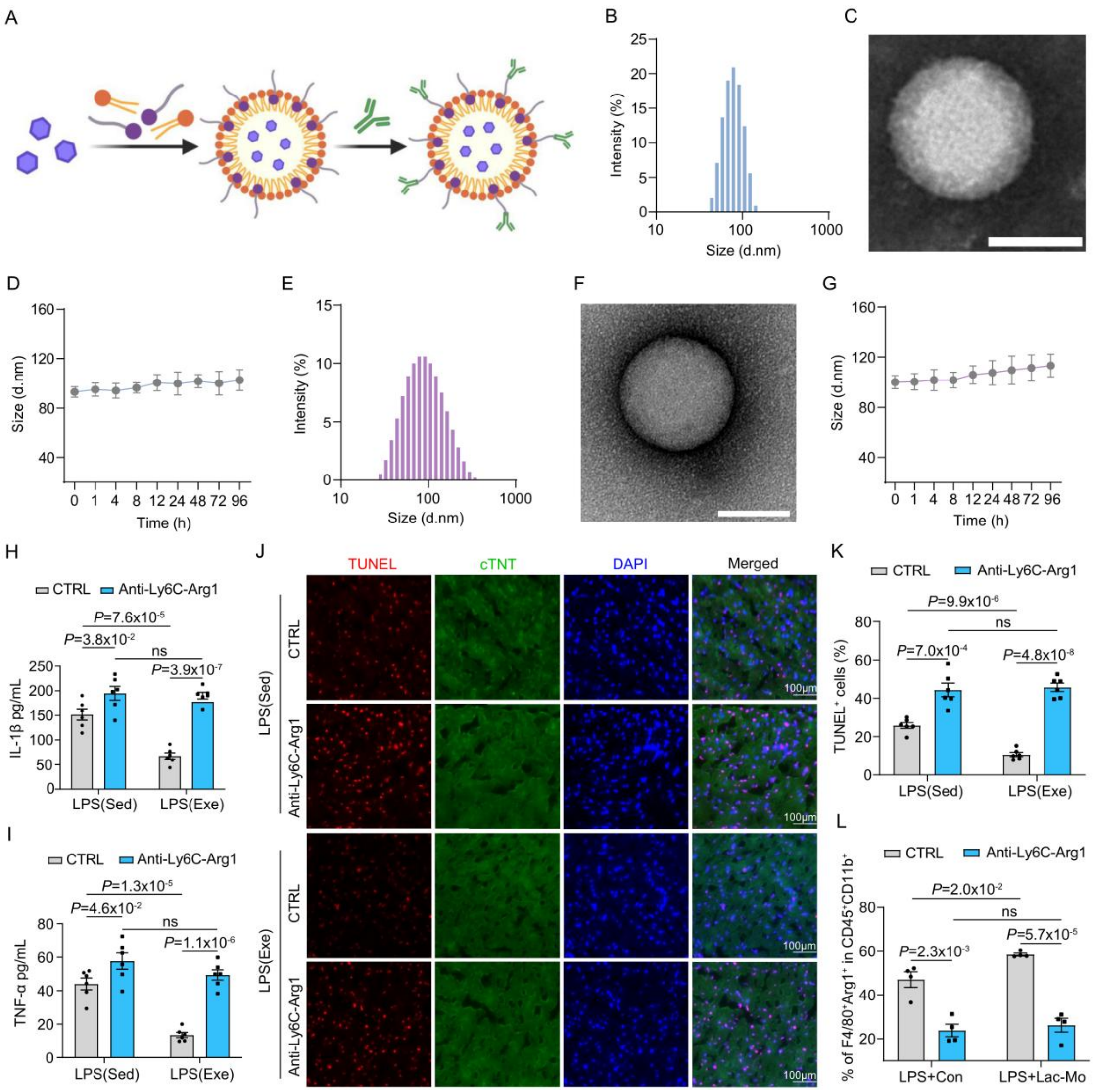

Figure S6. The nano-inhibitor designed to specifically inhibit iNOS and Arg1 in monocytes.

A, Schematic diagram of the nano-inhibitor. Figure created in BioRender, Shang, M. (2025) p8qhowz.

B,E, Particle size distribution diagram of nanoparticles for iNOS (**B**) and Arg1 (**E**) inhibitors.

C,F, TEM micrograph of nanoparticles for iNOS (**C**) and Arg1 (**F**) inhibitors.

D,G, Temporal evolution of nanoparticle size for iNOS (**D**) and Arg1 (**G**) inhibitors.

H-I, ELISA analysis of IL-1β (**H**) and TNF-α (**I**) in serum from exercise (Exe) and sedentary (Sed) mice, treated with Anti-Ly6C-Arg1 or control (CTRL). The analysis was performed on 6 samples per group.

J-K, Representative images (**J**) and quantification of TUNEL (**K**) positive area in hearts 18 hours post LPS injection in Exe and Sed mice, treated with Anti-Ly6C-Arg1 or control (CTRL). The analysis was performed on 6 samples per group.

L, Percentage of F4/80^{+}Arg1^{+} cells in CD45^{+}CD11b^{+} populations in hearts 18 hours post LPS injection in Exe and Sed mice, treated with Anti-Ly6C-Arg1 or control (CTRL). The analysis was performed on 4 samples per group from 2 independent experiments.

Data are represented as mean ± SEM. Unpaired two-tailed t-test was used to determine the statistical significance. ns, not significant. Scale bar: 100nm (**C, F**), **100** µm (**J**). Source data for **H-I** and **K-L** are provided in the Source Data file.

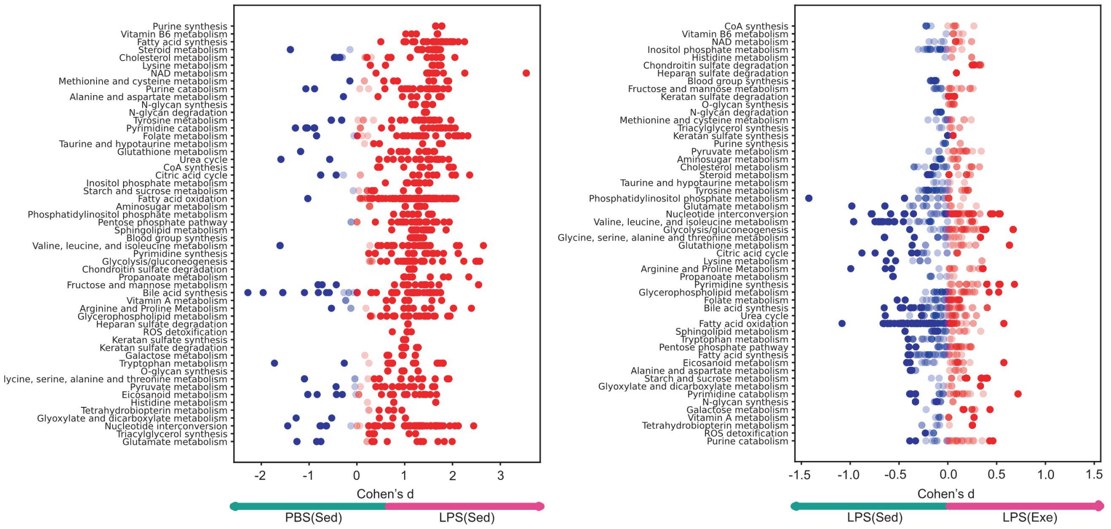

Figure S7. Differential activity of metabolic reactions.

Reactions (dots) are partitioned by Recon2 pathways and colored by the sign of their Cohen's d statistic.

Mø & Mo		Mø		Mo		Lyve1+ Mø		MHC II^{high} Mø		Ccr2+ Mø		Ly6c2^{high} Mo		Ly6c2^{low} Mo		iNOS+ Mo		
log2FC1	log2FC2	log2FC1	log2FC2	log2FC1	log2FC2	log2FC1	log2FC2	log2FC1	log2FC2	log2FC1	log2FC2	log2FC1	log2FC2	log2FC1	log2FC2	log2FC1	log2FC2	
0.56	0.10	0.73	0.09	0.29	0.12	0.47		0.96		0.66	0.22	0.27	0.18			0.44	0.17	Glycolysis / Gluconeogenesis
1.23	-0.29	1.31	-0.34	1.05	-0.24	0.87	-0.47	1.64	-0.24	1.44	-0.16	0.96	-0.15	1.39	-0.26	0.68		Citrate cycle (TCA cycle)
0.21	-0.05	0.46	-0.12	-0.14		0.43		0.57		0.27		-0.24	0.07	0.19		-0.40		Pentose phosphate pathway
0.31	0.22			0.78	0.21	-0.49					0.57	0.49	0.27	2.06				Pentose and glucuronate interconversions
3.44	0.14	2.87		2.84	0.29	1.21		3.46		2.47	0.29	3.28	0.36	0.25		3.03		Fructose and mannose metabolism
				-0.19		-0.49	-0.24	0.77		0.32	0.40	-0.23	0.16				0.45	Pyruvate metabolism
0.82	-0.25	0.60	-0.29	2.05	-0.20		-0.26	0.97	-0.37	1.01		1.34						Glyoxylate and dicarboxylate metabolism
-0.38	0.37	-0.58	0.49	-0.17	0.25	-0.35	0.63	-0.74	0.39	-0.61	0.33		0.33	-0.28				Propanoate metabolism
-1.75	1.12	-1.09	0.67	-4.41	3.46	-0.94	0.70	-1.12	0.63	-1.49		-4.46	3.52	-2.46	1.52			Inositol phosphate metabolism
0.33	0.04	0.42		0.16	0.05	0.25		0.58		0.39	0.08	0.13	0.10	0.19			0.10	Oxidative phosphorylation
	-0.35	0.12	-0.34	-0.29	-0.39	0.17	-0.28		-0.49		-0.29	-0.45	-0.34		-0.54	-0.69		Nitrogen metabolism
1.12	-0.19	0.92	-0.21	2.82		1.17		0.93	-0.33	0.45	-0.30	1.93		0.52				Sulfur metabolism
-0.39	0.34	-1.16	0.62		0.23	-1.18	0.55	-3.08	2.08	-0.41	0.48	-0.17	0.27					Fatty acid biosynthesis
2.22	-2.50	1.86	-3.85	3.14	-1.68		-2.68	2.84	-2.59	4.09	-1.59	1.34	-1.20	0.94			-0.64	Fatty acid degradation
0.85	-0.14	0.91		0.70	-0.17	1.38		0.66		0.81		0.87						Steroid hormone biosynthesis
0.72	-0.14	0.66	-0.17	1.11		0.50	-0.18	0.87	-0.18	0.49		0.76		4.17				Glycerophospholipid metabolism
0.37	-0.14		-0.20	0.63			-0.25			0.76		0.72	-0.12	0.39		1.09		Ether lipid metabolism
	-1.94		-1.66		-2.41		-0.88		-1.18		-3.75		-1.59		-0.66		-1.43	Sphingolipid metabolism
-1.48	0.62	-1.60	0.55	-1.35	0.70	-1.49	0.63	-1.99		-1.40	0.69	-1.33	0.70	-1.41	0.65	-1.42	0.72	Arachidonic acid metabolism
0.37	-0.18	0.62	-0.25			0.59		0.87			-0.40							alpha-Linolenic acid metabolism
1.66	0.42	1.72	0.49	1.54	0.34	4.80	0.64	0.99	0.45	2.59		3.17	0.38					Biosynthesis of unsaturated fatty acids
0.19	-0.31	0.31	-0.31		-0.30	0.32	-0.20		-0.50	0.42			-0.16		-0.48		-0.48	Alanine, aspartate and glutamate metabolism
-0.21	-0.74		-0.53	-0.37	-1.12	0.44	-0.35	-0.42		-1.07	-2.73		-3.84		-0.31		-0.41	Glycine, serine and threonine metabolism
0.52	1.02	0.38	1.07	0.91	0.95	5.87	1.03	-0.66	1.17		0.96	1.57	1.29	-0.55			0.68	Valine, leucine and isoleucine degradation
1.17	-1.67	1.21	-1.77	1.05	-1.56	0.59	-1.70	1.59	-1.86	2.01	-1.67	1.37	-1.44		-1.59		-1.90	Lysine degradation
0.35	-0.23	0.33	-0.32	0.36	-0.14	0.27	-0.27		-0.46	0.58		0.19		0.64	-0.56			Arginine biosynthesis
-2.17	0.37	-3.28		-1.08	0.37	-5.87		-3.54		-1.42	0.54	-0.95	0.47	-1.15				Arginine and proline metabolism
4.00	0.55	2.30	0.31	0.53	1.31	2.10	0.47	2.55		2.10		1.15	1.24	-1.18			1.20	Tyrosine metabolism
1.29	0.89	1.45	0.76	1.10	1.12	2.21	0.87	1.14	0.86	1.13	0.65		1.18				1.00	Phenylalanine metabolism
1.52		1.67		1.28		4.15		1.12		0.94		1.69		0.73		1.42		Tryptophan metabolism
0.87		0.90	-0.08	0.85		0.89		0.91		0.86		0.77	0.09	0.97		0.87		Selenocompound metabolism
	0.08	0.17		-0.32	0.09			0.36			0.28	-0.38	0.11	-0.23				Glutathione metabolism
0.91	-0.45	0.84	-0.44	1.16	-0.46		-0.30	1.24	-0.68	1.64		1.51	-0.25					N-Glycan biosynthesis
0.65	0.21	0.43	0.26	1.11	0.16	0.53	0.27	0.32	0.25	0.60	0.03	0.99	0.19	1.27		1.05		Mucin type O-glycan biosynthesis
0.19	-0.07	0.16		0.17	-0.09					0.23	-0.17	0.15	-0.07	0.15	-0.10			Mannose type O-glycan biosynthesis
0.44		0.63				0.79		0.66										Other types of O-glycan biosynthesis
0.54	-0.21	0.54	-0.22	0.54	-0.19	0.57	-0.22	0.55	-0.21	0.47	-0.21	0.53	-0.19	0.50		0.52		Glycosaminoglycan biosynthesis - chondroitin sulfate / dermatan sulfate
0.17	-0.12	0.16	-0.11	0.17	-0.12	0.09	-0.13	0.16		0.18			-0.08	0.40	-0.17			Glycosaminoglycan biosynthesis - heparan sulfate / heparin
		-0.61	0.28	0.43		-0.58		-0.63		-0.35		0.58						Glycosaminoglycan biosynthesis - keratan sulfate
-2.02		-2.11		-1.17		-2.45		-1.82		-1.98		-1.68		-0.57		-0.53		Glycosaminoglycan degradation
0.29	-0.23	0.29	-0.22	0.28	-0.25	0.33	-0.22	0.28	-0.21	0.20	-0.21	0.24	-0.26	0.30	-0.25		-0.23	Glycosylphosphatidylinositol (GPI)-anchor biosynthesis
1.26	-0.16	1.35	-0.20	1.10	-0.11	1.44	-0.28	1.63		0.78		1.12		0.73				Glycosphingolipid biosynthesis - lacto and neolacto series
1.97	-0.09	1.07	-0.12	6.37		0.49	-0.22	1.18		3.17		5.17		2.91	-0.21	0.47		Riboflavin metabolism
0.30	-0.19	0.34	-0.20	0.22	-0.17	0.26	-0.20	0.39	-0.18	0.36	-0.17	0.20	-0.12	0.28	-0.23		-0.20	Pantothenate and CoA biosynthesis
-5.38				-5.39	1.39	-1.17						-5.89	1.89	-1.89		-4.04		Folate biosynthesis
0.29	-0.08	0.31		0.22	-0.09	0.35	-0.15	0.30				0.08	-0.09	0.54	-0.12			One carbon pool by folate
0.60	-0.18	0.75	-0.19	0.36	-0.16	0.98	-0.20	0.67	-0.17	0.50	-0.21	0.40	-0.18	0.24			-0.20	Retinol metabolism
0.51	0.28	0.41	0.24	1.30	0.37		0.21	0.71		0.88		0.69	0.33	0.27	2.40			Porphyrin and chlorophyll metabolism
0.37	-0.21	0.50	-0.25	0.14	-0.16	0.56	-0.26	0.44	-0.18	0.48	-0.33		-0.21	0.16			-0.30	Ubiquinone and other terpenoid-quinone biosynthesis
-1.08	0.48	-1.54	0.62	-0.56	0.38	-1.87	1.04	-1.23		-1.43	0.67	-0.65	0.45					Terpenoid backbone biosynthesis
1.04	-0.22	0.90	-0.20	1.47	-0.26	1.08	-0.25	0.92	-0.15	0.52	-0.19	1.92	-0.21	0.84	-0.32			Metabolism of xenobiotics by cytochrome P450
0.93	-0.27	0.71	-0.25	1.54	-0.31	0.84	-0.30	0.74	-0.19	0.41	-0.24	2.11	-0.27	0.81	-0.31	0.67	-0.32	Drug metabolism - cytochrome P450

Figure S8. Heatmap of metabolic pathway activity differences among monocyte and macrophage subsets.

The heatmap displays differences in pathway activity among different groups of monocytes and macrophages subsets based on scMetablism analysis. Only pathways with significant changes exhibit FC values. FC1 represents the fold change between LPS (Sed) and PBS (Sed) group, while FC2 represents the fold change between LPS (Exe) and LPS (Sed) group. Source data is provided in the Source Data file.

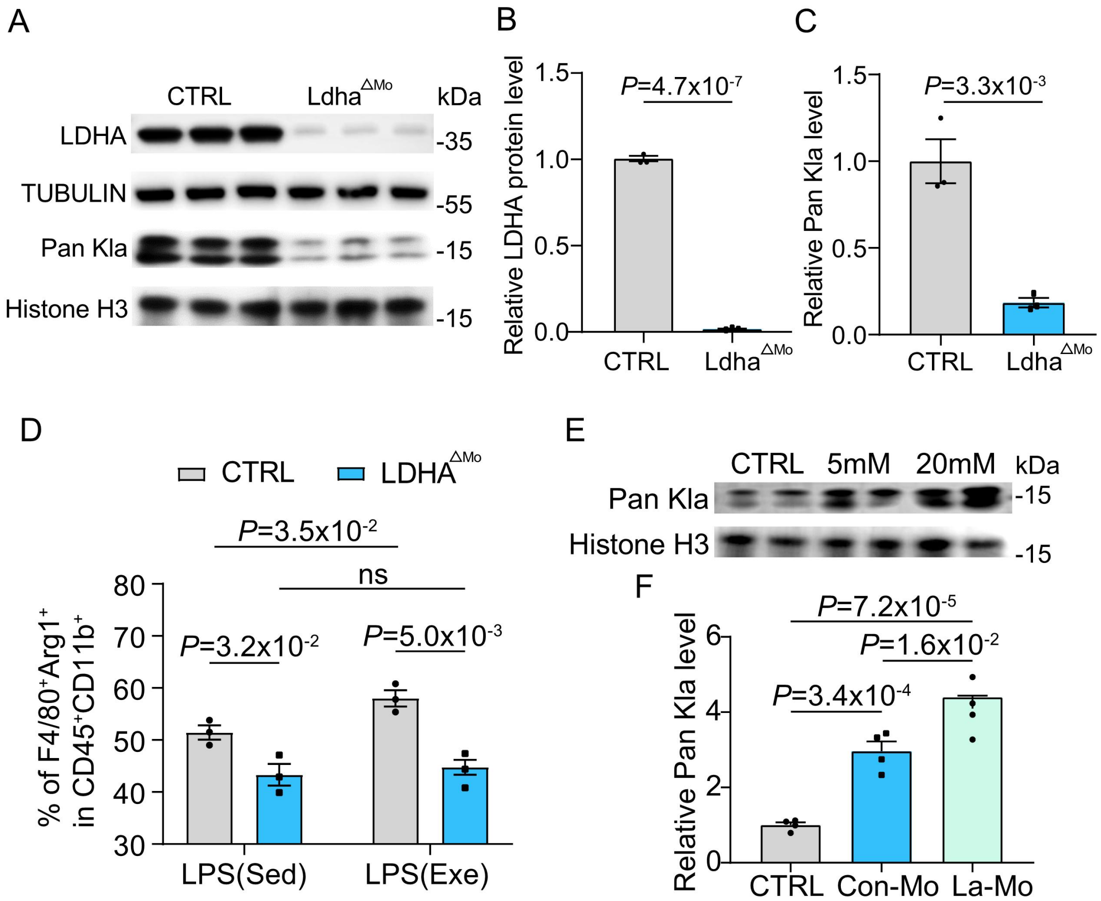

Figure S9. Pan Kla and H3K18 levels in monocytes in *Ldha*$^{\Delta Mo}$ mice.

A-C, LDHA knockout efficiency and Pan kla in monocytes from *Ldha*$^{\Delta Mo}$ mice (CTRL, n=3; *Ldha*$^{\Delta Mo}$, n=3) detected by western blot. β-Tubulin was used as loading control. Numbers represent densitometric fold change relative to β-Tubulin. The analysis was performed on 3 samples per group.

D, The percentage of F4/80$^+$ Arg1$^+$ cells in CD45$^+$ CD11b$^+$ populations in *Ldha*$^{\Delta Mo}$ or control mice 18 hours post-LPS injection, with or without exercise. The analysis was performed on 3 samples per group.

E-F, Western blot for Pan Kla in monocytes incubated with 5mM or 20 mM of sodium lactate for 24 hours. Histone H3 used as an internal control, with densitometric fold changes relative to Histone H3 indicated. The analysis was performed on 4 samples per group.

All experiments show representative values from three independent experiments. Data are represented as mean ± SEM. Unpaired two-tailed t-test was used to determine the statistical significance. ns, not significant. Source data for **A-F** are provided in the Source Data file.

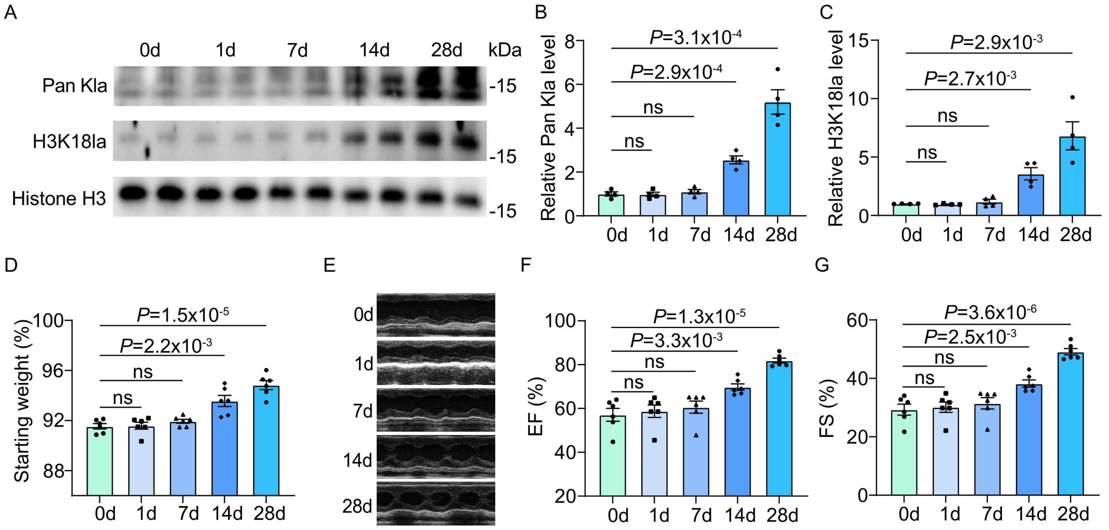

Figure S10. A time-course analysis of exercise-induced histone lactylation in monocytes and its protective effects on cardiac function during SICM.

A-C, Western blot for Pan Kla and H3K18la in monocytes from Exe mice 18h post injection with LPS. Exercise duration gradients were set at 0 days, 1 day, 7 days, 14 days, and 28 days. Histone H3 used as an internal control, with densitometric fold change relative to Histone H3 indicated. The analysis was performed on 4 samples per group.

D, Percent change in body weight measured 18 hours after i.p. injection of LPS. Exercise duration gradients were set at 0 days, 1 day, 7 days, 14 days, and 28 days. The analysis was performed on 6 samples per group.

E-G, Representative echocardiography images (**E**) and quantification of EF% (**F**), FS % (**G**). The analysis was performed on 6 samples per group.

Data are represented as mean ± SEM. Unpaired two-tailed t-test was used to determine the statistical significance. ns, not significant. Source data for **A-D, F-G** are provided in the Source Data file.

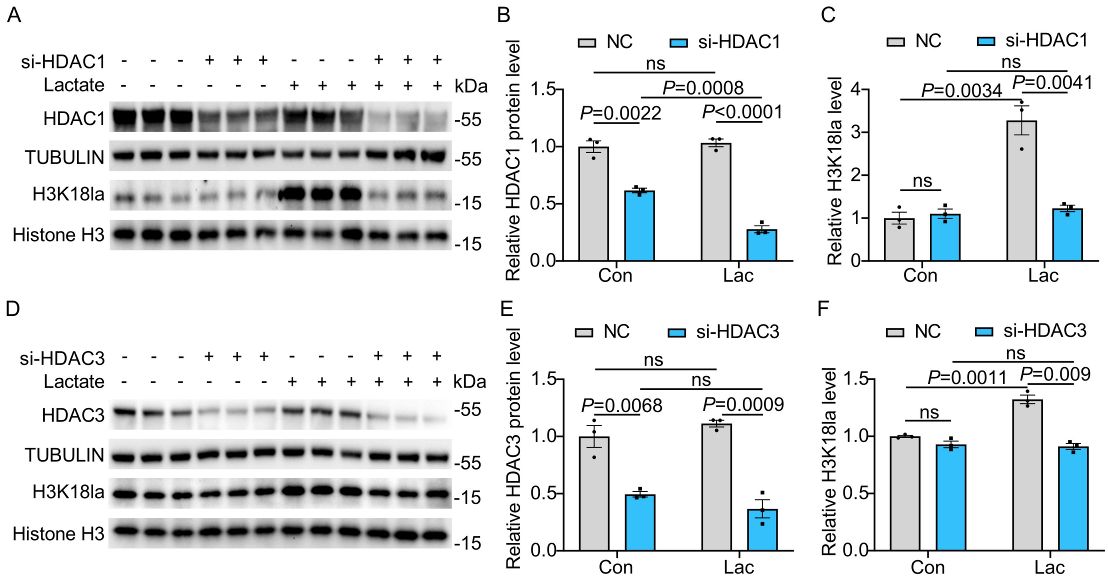

Figure S11. Exercise-induced H3K18 histone lactylation in monocytes is independent of HDAC1 and HDAC3.

A-C, Western blot for H3K18la in monocytes from mice subjected to HDAC1 knockdown using si-HDAC1, treatment with 20 mM sodium lactate for 24 hours, and their respective control groups. Histone H3 was used as an internal control, with densitometric fold change relative to Histone H3 indicated. The analysis was performed on 3 samples per group.

D-F Western blot for H3K18la in monocytes from mice subjected to HDAC3 knockdown using si-HDAC3, treatment with 20 mM sodium lactate for 24 hours, and their respective control groups. Histone H3 used as an internal control, with densitometric fold change relative to Histone H3 indicated. The analysis was performed on 3 samples per group.

All experiments show representative values from at least 2 independent experiments. Data are represented as mean ± SEM. Unpaired two-tailed t-test was used to determine the statistical significance. ns, not significant. Source data for **A-F** are provided in the Source Data file.

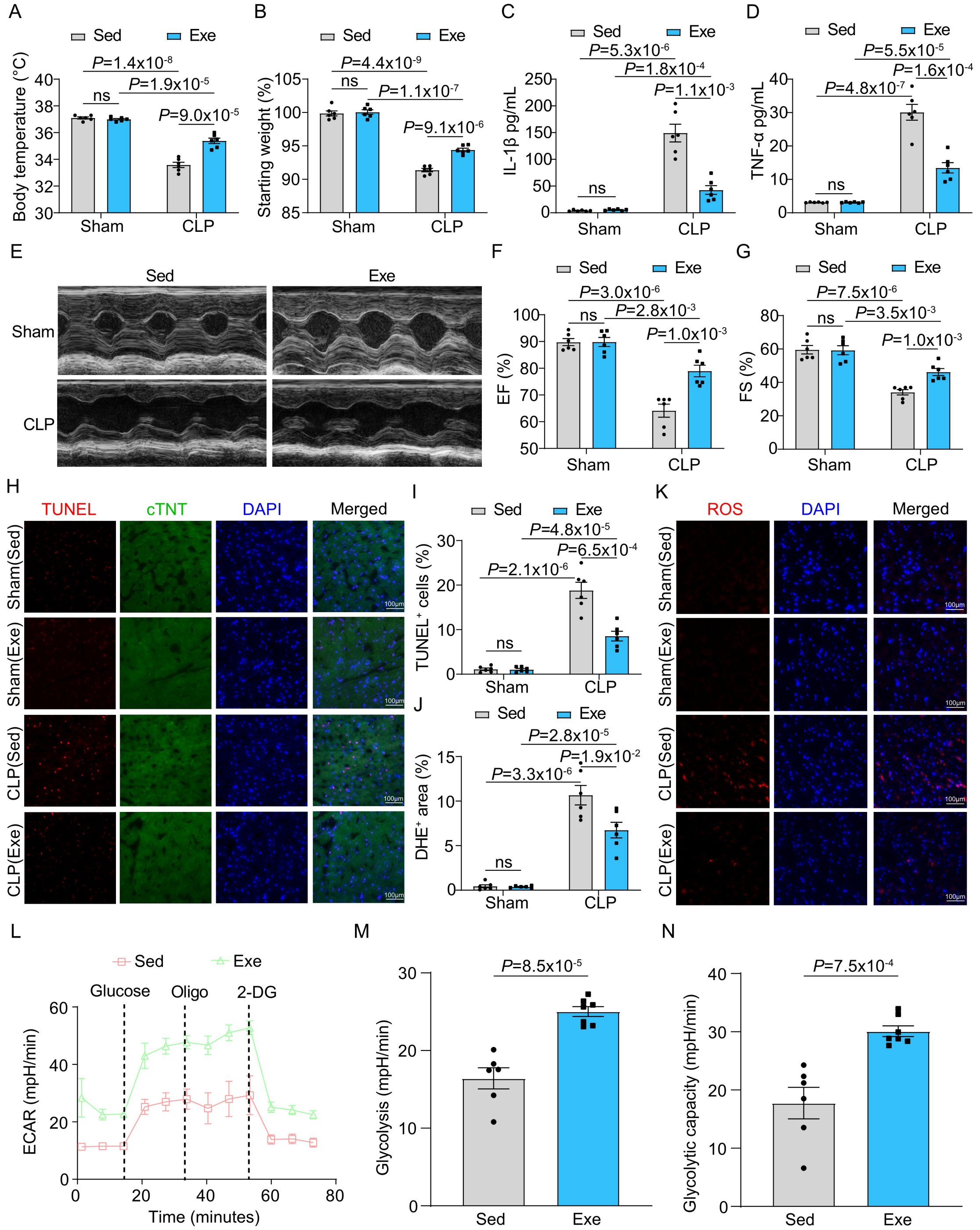

Figure S12. Regular physical exercise safeguards cardiac function in CLP induced SICM.

A-B, Body temperature (**A**) and percent change in body weight (**B**) of mice 24 hours post cecal ligation and puncture (CLP). The analysis was performed on 6 samples per group.

C-D, ELISA analysis of IL-1β (**C**) and TNF-α (**D**) levels in serum from Sed and Exe mice 24 hours post CLP. The analysis was performed on 6 samples per group.

E-G, Representative echocardiography images (**E**), EF% (**F**) and FS% (**G**) of Sed and Exe mice 24 hours post CLP. The analysis was performed on 6 samples per group.

H-**K**, Representative images and quantification of TUNEL (**H,I**) and DHE (**J,K**) in heart sections 24 hours post CLP in Sed and Exe mice. The analysis was performed on 6 samples per group.

L-N, Extracellular acidification rate (ECAR) in monocytes isolated from Exe or Sed mice (Sed, n=6; Exe, n=7).

Data are represented as mean ± SEM. Unpaired two-tailed t-test was used to determine the statistical significance. ns, not significant. Scale bar: 100µm (**H, K**). Source data for **A-D, F-G, I-J and L-N** are provided in the Source Data file.

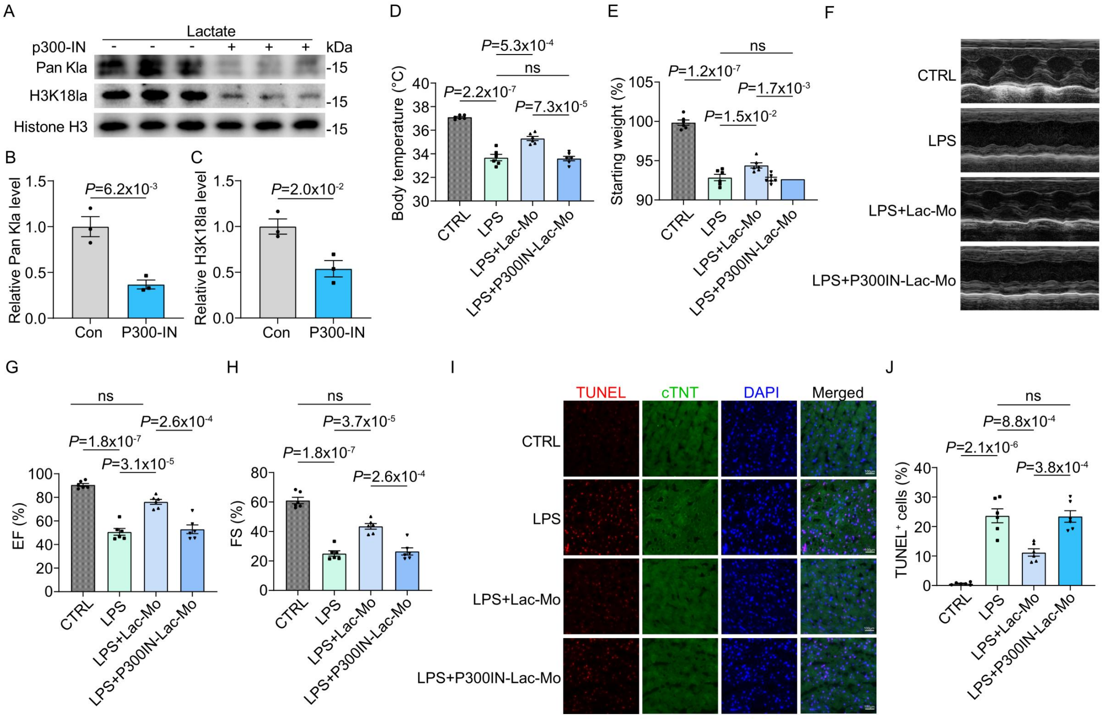

Figure S13. Histone lactylation at H3K18, with p300 as the writer, is essential for preserving cardiac function in SICM.

A-C. Western blot for Pan Kla and H3K18la in monocytes from mice. Histone H3 used as the internal control, with densitometric fold change relative to tubulin and Histone H3 indicated. The analysis was performed on 3 samples per group from 3 independent experiments.

D-E, Body temperature (**D**) and percent change in body weight (**E**) of SICM mice after lactate-educated monocyte reinfusion, with or without P300 inhibitor. The analysis was performed on 6 samples per group.

F-H, Representative echocardiography images (**F**), EF% (**G**) and FS% (**H**) in SICM mice after lactate-educated monocyte reinfusion with or without P300 inhibitor. The analysis was performed on 6 samples per group.

I-J, Representative images (**I**) and quantification of TUNEL (**J**) for heart sections of SICM mice after lactate-educated monocyte reinfusion, with or without P300 inhibitor. The analysis was performed on 6 samples per group.

Data are represented as mean ± SEM. Unpaired two-tailed t-test was used to determine the statistical significance. ns, not significant. Scale bar: 100µm (**I**). Source data for **A-E, G-H and J** are provided in the Source Data file.

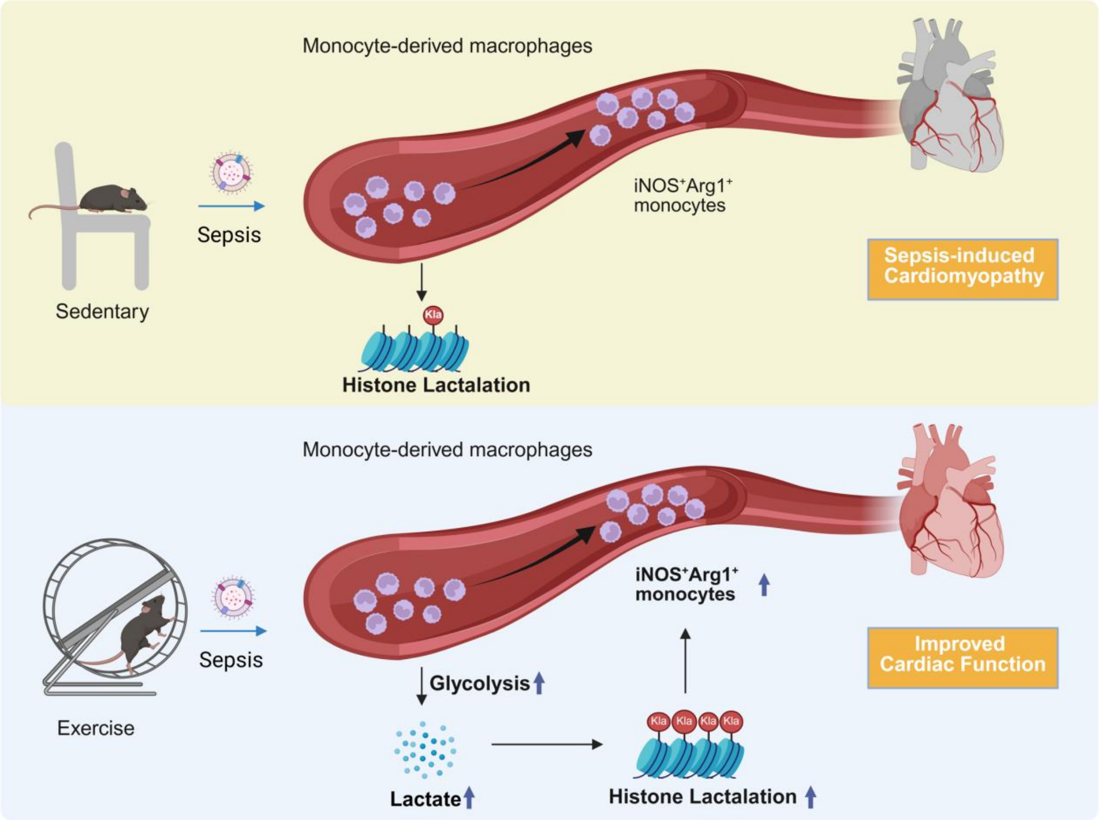

Figure S14. Figure scheme.

An active lifestyle protects cardiac function in sepsis-induced cardiomyopathy (SICM). Exercise increased the infiltration of iNOS$^+$ Arg1$^+$ subsets into the heart during SICM, representing a unique subpopulation of monocyte-derived cardiac macrophages identified in this study. These iNOS$^+$ Arg1$^+$ subsets express both pro-inflammatory and pro-reparative genes. The inhibition of either pro-inflammatory iNOS or pro-reparative Arg1 in monocytes and derived macrophages impairs the exercise-induced preservation of cardiac function. Following enhanced pro-inflammatory immune responses to combat early-stage infections, exercise accelerates the transformation of cardiac macrophages from a pro-inflammatory to a wound-healing state. This is achieved by enhancing glycolysis in monocytes, leading to increased lactate production, which boosts histone lactylation at H3K18, thereby restoring cardiac immune homeostasis and preserving cardiac function in SICM. The adoptive transfer of monocytes with high histone lactylation mimics the cardioprotective effects of exercise in SICM mice. Figure created in BioRender, Shang, M. (2025) leivg1v.

Table S1. Basic sequencing and quality control statistics of the scRNA-seq data.

	Estimates	PBS(Sed)1	PBS(Sed)2	PBS(Sed)3	LPS(Sed)1	LPS(Sed)2	LPS(Sed)3	LPS(Exe)1	LPS(Exe)2	LPS(Exe)3	LPS(Exe)4	Total
Basic sequencing and mapping statistics a)	Estimated Number of Cells	4,906	6,319	2,992	2,226	4,139	4,612	3,317	3,402	9,777	15,427	57,117
	Mean Reads per Cell	71,222	49,801	122,027	205,166	106,449	87,571	126,037	106,414	32,505	31,675	
	Median Genes per Cell	1,414	1,324	1,384	1,882	2,823	2,459	2,612	2,617	2,028	1,577	
	Total Genes Detected	18,431	18,853	17,988	18,385	19,298	19,136	18,784	18,588	19,482	19,946	
	Median UMI Counts per Cell	4,339	3,851	3,874	6,433	11,813	9,969	10,143	10,044	6,849	4,463	
	Valid Barcodes	98.4%	98.4%	98.4%	97.5%	97.6%	97.7%	97.6%	97.6%	96.7%	97.0%	
	Reads Mapped to Genome	94.6%	95.6%	95.0%	93.6%	92.6%	92.5%	92.6%	95.8%	95.5%	94.8%	
Statistics after QC in Seurat b)	Number of Cells	3,993	5,527	2,492	1,935	3,464	3,919	2,766	2,960	8,842	14,573	50,471
	Cd45+ Cells	3,376	4,340	1,849	1,209	3,139	3,564	2,460	2,690	7,547	10,718	40,892
	Total Expressed Genes	16,026	16,410	15,458	15,744	16,790	16,632	16,211	16,148	17,059	17,545	19,184

a) The basic sequencing and mapping statistics were the results acquired from CellRanger output results. b) The statistics after QC in Seurat were obtained from filtering out the cells that expressed less than 200 or more than 5000 unique genes or more than 20% of reads mapping to mitochondria in Seurat.

Table S2. Basic clinical information of SICM patients and healthy donors.

	Healthy	SICM
Gender	Male(n=4); Female(n=5)	Male(n=6); Female(n=4)
Age	32.33 ± 6.13	43.8 ± 9.83
SOFA score	0	5.0 ± 2.1

Age and Sequential organ failure assessment (SOFA) score are shown as the mean ± SD.

Table S3. Basic clinical information of volunteers with sedentary and active lifestyles.

	Sedentary	Active
Gender	Male(n=4); Female(n=2)	Male(n=4); Female(n=2)
Age	24.33 ± 1.63	23.83 ± 1.94

Age is shown as the mean ± SD.
