# Supplementary material for: Exercise-induced histone lactylation in monocyte-derived macrophages restores cardiac immune homeostasis and function in sepsis-induced cardiomyopathy
Source: Nat Commun. 2025 Dec 15;17:756. doi: 10.1038/s41467-025-67443-8 (PMC12819526; doi:10.1038/s41467-025-67443-8)
Supplement: Supplementary file 2 — Reporting Summary [file 41467_2025_67443_MOESM2_ESM.pdf]

nature portfolio

Corresponding author(s): Min Shang; Guosheng Fu; Boxuan Ma; Junbin Qian

Last updated by author(s): Nov 11, 2025

Reporting Summary

Nature Portfolio wishes to improve the reproducibility of the work that we publish. This form provides structure for consistency and transparency in reporting. For further information on Nature Portfolio policies, see our Editorial Policies and the Editorial Policy Checklist.

Statistics

For all statistical analyses, confirm that the following items are present in the figure legend, table legend, main text, or Methods section.

n/a	Confirmed	
☐	☒	The exact sample size (*n*) for each experimental group/condition, given as a discrete number and unit of measurement
☐	☒	A statement on whether measurements were taken from distinct samples or whether the same sample was measured repeatedly
☐	☒	The statistical test(s) used AND whether they are one- or two-sided<br>*Only common tests should be described solely by name; describe more complex techniques in the Methods section.*
☐	☒	A description of all covariates tested
☐	☒	A description of any assumptions or corrections, such as tests of normality and adjustment for multiple comparisons
☐	☒	A full description of the statistical parameters including central tendency (e.g. means) or other basic estimates (e.g. regression coefficient) AND variation (e.g. standard deviation) or associated estimates of uncertainty (e.g. confidence intervals)
☐	☒	For null hypothesis testing, the test statistic (e.g. *F*, *t*, *r*) with confidence intervals, effect sizes, degrees of freedom and *P* value noted<br>*Give P values as exact values whenever suitable.*
☒	☐	For Bayesian analysis, information on the choice of priors and Markov chain Monte Carlo settings
☒	☐	For hierarchical and complex designs, identification of the appropriate level for tests and full reporting of outcomes
☐	☒	Estimates of effect sizes (e.g. Cohen's *d*, Pearson's *r*), indicating how they were calculated

Our web collection on statistics for biologists contains articles on many of the points above.

Software and code

Policy information about availability of computer code

Data collection	Microscopy: CellSens Dimension 3.1; Western blot: GE Amersham Imager 600; RT-qPCR: QuantStudio Flex (Thermo).<br>Cell sorting: Beckman MoFlo Astrios EQ Software Version 6.3.
Data analysis	Imaging: CellSens Dimension Desktop 3.1 and Image J 1.4.3; GraphPad Prism 9; Morphometry: ImageJ 1.47; Cell sorting: FlowJo Software Version 10.5.3.

For manuscripts utilizing custom algorithms or software that are central to the research but not yet described in published literature, software must be made available to editors and reviewers. We strongly encourage code deposition in a community repository (e.g. GitHub). See the Nature Portfolio guidelines for submitting code & software for further information.

Data

Policy information about availability of data

All manuscripts must include a data availability statement. This statement should provide the following information, where applicable:

- Accession codes, unique identifiers, or web links for publicly available datasets
- A description of any restrictions on data availability
- For clinical datasets or third party data, please ensure that the statement adheres to our policy

The sequencing data generated in this study were deposited at the National Center for Biotechnology Information’s Sequence Read Archive (SRA) under accession

numbers RJNA1171952 and PRJNA1300856. Source Data are provided for all experiments. Other data that support the findings of this study are available on request from the corresponding author upon reasonable request.

Research involving human participants, their data, or biological material

Policy information about studies with human participants or human data. See also policy information about sex, gender (identity/presentation), and sexual orientation and race, ethnicity and racism.

Reporting on sex and gender	The gender of volunteers in this study is provided in Table 2 and Table 3.
Reporting on race, ethnicity, or other socially relevant groupings	We didn't report on race, ethnicity,or other socially relevant groupings in this study.
Population characteristics	To obtain human peripheral blood monocytes for further RNA sequencing, a total of 19 participants were enrolled in this study, including 10 sepsis patients and 9 non-sepsis controls. Eligible participants met the following criteria: aged between 18 and 85 year. For sepsis cases, fulfillment of the Sepsis-3 definition of sepsis, with concurrent evidence of myocardial injury. Myocardial injury was defined as either elevated high-sensitivity troponin-I (hs-cTnI > 0.04 ng/mL) or echocardiographic evidence of acute left ventricular systolic dysfunction, specifically a left ventricular ejection fraction (LVEF) < 50% with a reduction of ≥ 10% in LVEF. For non-sepsis controls, individuals without a diagnosis of sepsis or evidence of myocardial injury were included, matched for basic demographic characteristics where applicable. To obtain human peripheral blood monocytes for histone lactalytion, a total of 12 volunteers were enrolled in this study, including 6 volunteers with regular exercise habits, defined as at lease 30 minutes exercise three times per week, and 6 volunteers with less than one session per week.
Recruitment	We collected blood samples with consent from healthy donor and sepsis patients with sepsis-induced cardiomyopathy at the hospital. In addition, a total of 12 volunteers were enrolled in this study, including 6 volunteers with regular exercise habits, defined as at least 30 minutes of exercise three times per week, and 6 volunteers with less than one session per week. All donors provided informed consent.
Ethics oversight	All experimental protocols using patient blood samples were approved by the Ethics Committee of Sir Run Run Shaw Hospital, School of Medicine, Zhejiang University (Approve number: 2024-0409, 2025-0443).

Note that full information on the approval of the study protocol must also be provided in the manuscript.

Field-specific reporting

Please select the one below that is the best fit for your research. If you are not sure, read the appropriate sections before making your selection.

☒ Life sciences ☐ Behavioural & social sciences ☐ Ecological, evolutionary & environmental sciences

For a reference copy of the document with all sections, see nature.com/documents/nr-reporting-summary-flat.pdf

Life sciences study design

All studies must disclose on these points even when the disclosure is negative.

Sample size	Sample sizes for all experiments were chosen based on our previous experience (Qinfeng, Li. et al. PKM1 Exerts Critical Roles in Cardiac Remodeling Under Pressure Overload in the Heart. Circulation. 2021 Aug 31;144(9):712-727.)
Data exclusions	A limited number of experiments that failed quality control (e.g. differing from the standard experimental procedures or accidentally influenced by unpredictable events) have been excluded from the study. No data were excluded from the showed experiments.
Replication	Experiments were successfully repeated at least 3 times. Independent experiments were performed in a blinded fashion to assure the reproducibility of the experimental findings.
Randomization	All samples involved were allocated into experimental groups randomly.
Blinding	Investigators were blinded to group allocation during data collection and analysis.

Reporting for specific materials, systems and methods

We require information from authors about some types of materials, experimental systems and methods used in many studies. Here, indicate whether each material, system or method listed is relevant to your study. If you are not sure if a list item applies to your research, read the appropriate section before selecting a response.

Materials & experimental systems

n/a	Involved in the study
☐	☒ Antibodies
☒	☐ Eukaryotic cell lines
☒	☐ Palaeontology and archaeology
☐	☒ Animals and other organisms
☒	☐ Clinical data
☒	☐ Dual use research of concern
☒	☐ Plants

Methods

n/a	Involved in the study
☒	☐ ChIP-seq
☐	☒ Flow cytometry
☒	☐ MRI-based neuroimaging

Antibodies

Antibodies used

Western blot: anti-Pan Kla (1:2000, PTM1401, PTM Bio); anti-Arg1 (1:1000, ab133543, Abcam); anti-iNOS (1:1000, ab15323, Abcam); anti-Histone H3 (1:1000,ab176842, Abcam); anti-β-tubulin (1:5000,10094, Proteintech); anti-LDHA (1:1000, ab52488, Abcam); anti-H3K18la (1:1000, PTM-1427RM, PTM Bio). Lactyl-Histone Antibody Sampler Kit (PTM-7093, PTM Bio); anti-P300 (1:500, 20695-1, Proteintech); anti-HDAC1(1:1000, 10197-1, Proteintech); anti-HDAC2 (1:5000, 12922-3, Proteintech); anti-HDAC3 (1:1000, 10255-1, Proteintech).
Histology and immunostainings: anti-CD68 (1:250, MCA1957GA, BioRad), anti-Arg1 (1:100, ab133543, Abcam), anti-iNOS (1:200, ab15323, Abcam), anti-Pan Kla (1:100, PTM1401, PTMBio), anti-cTnT (1:200, 68300, Proteintech), Alexa Fluor 488 Goat anti-Rabbit (Invitrogen, A-11008), Alexa Fluor 555 Goat anti-Rabbit (Invitrogen, A-21428), Alexa Fluor 647 Donkey anti-Rat (Invitrogen, A-21247), Alexa Fluor 488 Donkey anti-Mouse (Invitrogen, A-21202), Alexa Fluor 555 Donkey anti-Rabbit (Invitrogen, A-31572), Goat anti-rabbit DyLight 405 (Invitrogen, SA5-10044).

Validation

anti-Pan Kla (1:2000, PTM1401, PTM Bio):
1.Fei X, et al. p53 lysine-lactylated modification contributes to lipopolysaccharide-induced proinflammatory activation in BV2 cell under hypoxic conditions[J]. Neurochemistry International, 2024.
2.Zhou J, et al. Astrocytic LRP1 enables mitochondria transfer to neurons and mitigates brain ischemic stroke by suppressing ARF1 lactylation[J]. Cell Metabolism, 2024.
3.Zhang X, et al. Lactate drives epithelial-mesenchymal transition in diabetic kidney disease via the H3K14la/KLF5 pathway[J]. Redox Biology, 2024.
4.Wu S, et al. H3K18 lactylation accelerates liver fibrosis progression through facilitating SOX9 transcription[J]. Experimental Cell Research, 2024.

anti-Arg1 (1:1000, ab124917, Abcam)
5.Jing L, An Y, Cai T, et al. A subpopulation of CD146+ macrophages enhances antitumor immunity by activating the NLRP3 inflammasome[J]. Cell Mol Immunol, 2023, 20(8): 908-923. doi: 10.1038/s41423-023-01047-4.
6.Hu SH, Feng YY, Yang YX, et al. Amino acids downregulate SIRT4 to detoxify ammonia through the urea cycle[J]. Nat Metab, 2023, 5(4): 626-641. doi: 10.1038/s42255-023-00784-0.

anti-iNOS (1:1000, ab15323, Abcam)
7.Chen S, Yu Y, Xie S, et al. Local H2 release remodels senescence microenvironment for improved repair of injured bone[J]. Nat Commun, 2023, 14(1): 7783. doi: 10.1038/s41467-023-43618-z.
8.Kikuchi K, Otsuka S, Takada S, et al. 1,5-anhydro-D-fructose induces anti-aging effects on aging-associated brain diseases by increasing 5'-adenosine monophosphate-activated protein kinase activity via the peroxisome proliferator-activated receptor-γ co-activator-1α/brain-derived neurotrophic factor pathway[J]. Aging (Albany NY), 2023, 15(21): 11740-11763. doi: 10.18632/aging.205228.

anti-Histone H3 (1:1000, ab176842, Abcam)
9.Chakraborty C, Nissen I, Vincent CA, et al. Rewiring of the promoter-enhancer interactome and regulatory landscape in glioblastoma orchestrates gene expression underlying neurogliomal synaptic communication[J]. Nat Commun, 2023, 14(1): 6446. doi: 10.1038/s41467-023-41919-x.
10.Zhou D, Liu W, Zhang J, et al. Bellidifolin ameliorates isoprenaline-induced cardiac hypertrophy by the Nox4/ROS signalling pathway through inhibiting BRD4[J]. Cell Death Discov, 2023, 9(1): 279. doi: 10.1038/s41420-023-01563-2.
anti-β-tubulin (1:5000, 10094, Proteintech)
11.Huang D, Xu B, Liu L, et al. TMEM41B acts as an ER scramblase required for lipoprotein biogenesis and lipid homeostasis[J]. Cell Metab, 2021, 33(8): 1655-1670.e8. doi: 10.1016/j.cmet.2021.05.006.
12.Rosina M, Ceci V, Turchi R, et al. Ejection of damaged mitochondria and their removal by macrophages ensure efficient thermogenesis in brown adipose tissue[J]. Cell Metab, 2022, 34(4): 533-548.e12. doi: 10.1016/j.cmet.2022.02.016.

anti-LDHA (1:1000, ab52488, Abcam)
13.Zhang C, Liu L, Li W, et al. Upregulation of FAM83F by c-Myc promotes cervical cancer growth and aerobic glycolysis via Wnt/β-catenin signaling activation[J]. Cell Death Dis, 2023, 14(12): 837. doi: 10.1038/s41419-023-06377-9.
14.Zhang X, Zhong Y, Liu L, et al. Fasting regulates mitochondrial function through lncRNA PRKCQ-AS1-mediated IGF2BPs in papillary thyroid carcinoma[J]. Cell Death Dis, 2023, 14(12): 827. doi: 10.1038/s41419-023-06348-0.
anti-H3K18la (1:1000, PTM-1427RM, PTM Bio)
15.Zhang Y, et al. Virus-Induced Histone Lactylation Promotes Virus Infection in Crustacean[J]. Advanced Science, 2024.
16.Qiao J, et al. Histone H3K18 and Ezrin Lactylation Promote Renal Dysfunction in Sepsis-Associated Acute Kidney Injury[J]. Advanced Science, 2024.
17.Zhang Y, et al. Macrophage MCT4 inhibition activates reparative genes and protects from atherosclerosis by histone H3 lysine 18 lactylation[J]. Cell Reports, 2024.

Lactyl-Histone Antibody Sampler Kit (PTM-7093, PTM Bio)
18.Duan W, et al. Warburg effect enhanced by AKR1B10 promotes acquired resistance to pemetrexed in lung cancer-derived brain metastasis[J]. Journal of Translational Medicine, 2023.
19.Zhao SS, et al. Lactate regulates pathological cardiac hypertrophy via histone lactylation modification[J]. Journal of Cellular and Molecular Medicine, 2024.
20.Zhang HR, et al. Lactate-Induced Mitochondrial Calcium Uptake 3 Aggravates Myocardial Ischemia–Reperfusion Injury by Promoting Neutrophil Extracellular Trap Formation[J]. Research, 2025.
21.Chen LH, et al. AhR-mediated histone lactylation drives cellular senescence during benzo[a]pyrene-evoked chronic obstructive pulmonary disease[J]. Journal Of Hazardous Materials, 2025.
anti-P300 (1:500, 20695-1, Proteintech)
22.Yan M, Wang Q, Yang H, et al. The Paeonol of Total Glucosides of White Peony Regulates the Differentiation of CD4+Treg Cells through the EP300/Foxp3 Axis to Relieve Pulmonary Fibrosis in Mice[J]. Cell Biochem Biophys, 2025, 83(3): 3959-3970. doi: 10.1007/s12013-025-01770-x.
23.Cai Q, Zhao Q, Yang Q, et al. The Ku protein family regulates hyperglycemia-induced vascular endothelial cell inflammation by modulating P300 levels[J]. Exp Cell Res, 2025, 444(2): 114399. doi: 10.1016/j.yexcr.2024.114399.

anti-HDAC1 (1:1000, 10197-1, Proteintech)
24.Zhong S, Li L, Zhang YL, et al. Acetaldehyde dehydrogenase 2 interactions with LDLR and AMPK regulate foam cell formation[J]. J Clin Invest, 2019, 129(1): 252-267. doi: 10.1172/JCI122064.
25.Ma L, Yu L, Jiang BC, et al. ZNF382 controls mouse neuropathic pain via silencer-based epigenetic inhibition of Cxcl13 in DRG neurons[J]. J Exp Med, 2021, 218(12): e20210920. doi: 10.1084/jem.20210920.
anti-HDAC2 (1:5000, 12922-3, Proteintech)
26.Zhong S, Li L, Zhang YL, et al. Acetaldehyde dehydrogenase 2 interactions with LDLR and AMPK regulate foam cell formation[J]. J Clin Invest, 2019, 129(1): 252-267. doi: 10.1172/JCI122064.
27. Li X, Yuan B, Lu M, et al. The methyltransferase METTL3 negatively regulates nonalcoholic steatohepatitis (NASH) progression[J]. Nat Commun, 2021, 12(1): 7213. doi: 10.1038/s41467-021-27539-3.

anti-HDAC3 (1:1000, 10255-1, Proteintech)
28.Chen Z, Huo D, Li L, et al. Nuclear DEK preserves hematopoietic stem cells potential via NCoR1/HDAC3-Akt1/2-mTOR axis[J]. J Exp Med, 2021, 218(5): e20201974. doi: 10.1084/jem.20201974.

Animals and other research organisms

Policy information about studies involving animals; ARRIVE guidelines recommended for reporting animal research, and Sex and Gender in Research

Laboratory animals	Myeloid-specific Ldha knockout mice were obtained from GemPharmatech Co., Ltd. in Jiangsu. These mice were created using CRISPR/Cas9-mediated genome engineering by inserting two loxP sites flanking exon 3 of the Ldha gene, resulting in Ldha Flox/Flox mice. These were subsequently crossed with LysM-Cre mice, which specifically express Cre recombinase in myeloid cell lineages, to produce myeloid-specific Ldha knockout mice. Wild-type mice, 8-week-old male C57BL/6, were sourced from GemPharmatech Co., Ltd. in Nanjing and maintained in an SPF-grade animal facility. The mice were housed under controlled conditions with consistent temperature and humidity, provided with standard chow and water ad libitum. The light-dark cycle was set to 12 hours of light and 12 hours of darkness.
Wild animals	No wild animals were used for this study.
Reporting on sex	The study exclusively utilized male mice to minimize potential confounding effects of estrogen in cardiovascular disease research, as estrogen is well-documented to exert protective roles in cardiovascular pathophysiology.
Field-collected samples	No field-collected samples were used for this study.
Ethics oversight	All experimental animal procedures were approved by the Animal Care and Use Committee of the School of Medicine, Zhejiang University (Approve number: ZJU20230056).

Note that full information on the approval of the study protocol must also be provided in the manuscript.

Plants

Seed stocks	Not applicable.
Novel plant genotypes	Not applicable.
Authentication	Not applicable.

Flow Cytometry

Plots

Confirm that:

☒ The axis labels state the marker and fluorochrome used (e.g. CD4-FITC).

☒ The axis scales are clearly visible. Include numbers along axes only for bottom left plot of group (a 'group' is an analysis of identical markers).

☒ All plots are contour plots with outliers or pseudocolor plots.

☒ A numerical value for number of cells or percentage (with statistics) is provided.

Methodology

Sample preparation	The hearts were perfused with 20 mL of cold PBS to remove peripheral blood. The ventricles were minced into 1 mm cubes. Heart tissues were digested in a solution containning 2 mg/mL collagenase IV, 1 mg/mL dispase II, and 60 U/ml DNAse at 37 degrees Celsius for 40 minutes with shaking. After dissociation, the cell suspensions were filtered through a 40 µm strainer, followed by red blood cell lysis.
Instrument	MoFlo Astrios EQ (Beckman)
Software	Data collection: MoFlo Astrios Summit Software (6.3).<br>Data analysis: FlowJo v10.5.3.
Cell population abundance	Aroud 7% out of alive cells are CD45+ in the heart harvested from mice subjected to intraperitoneal injection of PBS.<br>Around 13% out of alive cells are CD45+ in the heart harvested from mice subjected to 3-month exercise training or untrained condition, followed by intraperitoneal injection of LPS.<br>In the $CD45^+CD11b^+$ cell population isolated from the hearts of untrained wild-type mice, approximately 48% exhibited the $F480^+Arg1^+$ phenotype following intraperitoneal LPS administration. This proportion increased to roughly 60% in mice subjected to 1-month exercise training. Conversely, selective deletion of LDHA in myeloid cells resulted in a reduction of this population to approximately 40%.
Gating strategy	Leukocytes were gated for FSC/SSC, CD45 positive/alive.<br>See enclosed file (file name: Extended Data Figure1.Gating strategy).<br>The gating strategy for F480+Arg1+ macrophages is based on FSC/SSC, CD45 positive, and CD11b positive, included in the Source Data file.

☒ Tick this box to confirm that a figure exemplifying the gating strategy is provided in the Supplementary Information.
